# A case of mixed-pattern calcifications in multinodular goiter associated with a benign diagnosis

**DOI:** 10.1016/j.ijscr.2019.05.061

**Published:** 2019-06-08

**Authors:** D. Bianchi, U. Morandi, A. Stefani, B. Aramini

**Affiliations:** Division of Thoracic Surgery, Department of Medical and Surgical Sciences for Children and Adults, University Hospital of Modena, Via Largo del Pozzo n. 71, 41124 Modena, Italy

**Keywords:** US, endoscopic ultrasound, CT, computed tomography, FNA, fine-needle aspiration, Calcified nodules, Calcifications, Malignant nodules, Benign lesion, Endoscopic ultrasound

## Abstract

**Background:**

Calcified nodules are often found in a goiter when performing an ultrasound of the neck. An analysis of different calcification patterns could contribute to discriminating between nodules with a lower malignancy risk and those with a higher malignancy risk. In certain cases, the results of a simple cytological analysis of a nodule are not specific enough to be completely trusted.

**Case presentation:**

We present the case of a goiter with a mixed calcification pattern, including a type of calcification that is strongly associated with a malignant diagnosis, for which a completely benign diagnosis was determined.

**Conclusion:**

We believe that further studies examining a combination of ultrasound (US) and computed tomography (CT) scan presentations of thyroid nodules need to be performed.

## Highlights

• Calcified nodules in a goiter are often found when performing an ultrasound of the neck.
• Analysis of different calcification patterns could contribute to discriminating between nodules.
• Mixed pattern calcifications is not so frequent and not interpreted.
• We believe that a better radiologic evaluation setting of thyroid nodules needs to be performed.

## 1 Background

Calcifications are a common ultrasonographic finding in thyroid nodules, and the assessment of calcification patterns can be useful in differentiating between benign and malignant nodules. Depending on their diameter and echogenic characteristics, thyroid calcifications are divided into two categories: microcalcifications (<2 mm in diameter) and macrocalcifications (>2 mm in diameter with a posterior acoustic shadow).

Microcalcification has been found to be strongly associated with papillary thyroid carcinoma; it is found in up to 40% of cases [1]. On the other hand, various patterns of macrocalcification, such as annular or “egg-shell” calcification, are less clearly associated with malignancy.

The role of fine-needle aspiration (FNA) for thyroid nodules with macrocalcifications is unclear due to a relatively high rate of false negatives and nondiagnostic cytologies [2].

Our aim is to present a case of multinodular goiter in which we found a mixed pattern of calcifications (both micro- and macrocalcifications), but eventually reached a benign diagnosis. This work has been reported in line with the SCARE criteria [3].

## 2 Case presentation

We present a case involving a 76-year-old female patient who was first seen by her doctor for problems and discomfort with deglutition. Upon an objective examination, the thyroid gland appeared to be mildly enlarged (more on the right side of the neck) with a hard consistency, but mobile relative to the underlying tissues. The only comorbidity was a mental illness.

An ultrasound (US) of the neck was immediately performed and revealed a multinodular retrosternal goiter, for which the larger nodule in the right lobe presented with a diameter of approximately 6 cm and complete ring calcification. The goiter extended into the upper mediastinum and was lying on the aortic arch.

**Fig. 1:**
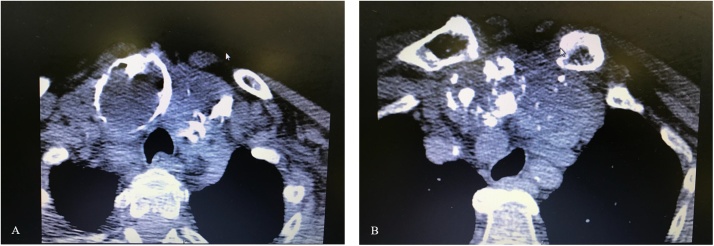
A. Calcified nodule with “egg-shell” pattern. B. Microcalcification spots in the same nodule.

Due to the goiter’s retrosternal configuration, we performed a CT scan of the neck and thorax. The scan showed the posterior dislocation of the epiaortic vessels and lateral dislocation and compression of the pharynx. The large nodule described previously based on US exhibited a mixed type of calcification; in fact, in the context of a large egg-shell calcification, multiple spots with microcalcifications were observed (Fig. 1A and B).

Given the notable dimensions of the goiter, its symptomatic disposition and the extreme calcification of the nodule, we chose to avoid an ultrasound-guided ago-biopsy due to the low probability of obtaining a good tissue sample and the consequent diagnosis, and in agreement with the patient’s endocrinologist, we decided to directly perform a standard thyroidectomy through a cervicotomy. The primary difficulty during the operation was the complete calcification of the entire right lobe, which was adherent to the trachea and vascular structures (Fig. 1A and B). However, no major bleeding or complications occurred during surgery.

**Fig. 2:**
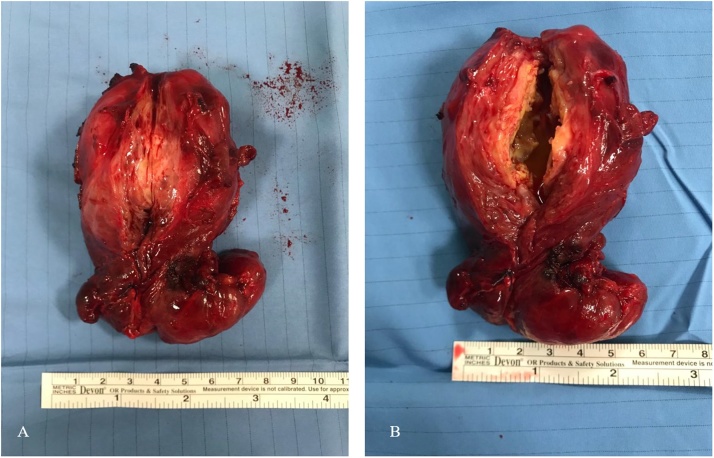
A. Macroscopically complete calcified nodule. B. Calcified rim when cut.

Macroscopically, the pathologist described a nodule with a weight of 130 g that was partially cavitated and extremely hard when cut (Fig. 2A and B).

A microscopic histological examination excluded the presence of atypical cells and revealed nodular hyperplasia in fibrotic areas.

The patient’s postoperative course did not involve any complications related to the surgical procedure, and she was discharged two days after the surgery.

## 3 Discussion and conclusion

It is commonly known that microcalcification patterns are associated with an elevated risk of papillary carcinoma findings in thyroid nodules, but that US findings of macrocalcifications in thyroid nodules are associated with a significantly lower malignancy risk (up to 27.6% of cases); in particular, Kim et al. reported that annular calcification was associated with the lowest risk of malignancy [1].

Nonetheless, for nodules with macrocalcifications, FNA has proven to be inaccurate and is therefore not helpful for discriminating between benign and malignant nodules, as observed by Dong Gyu Na, who described a nondiagnostic result in 65.1% of cases and inconclusive results in 67.1% of cases; a finding which leads us to the conclusion that US calcification patterns should be studied more to assess the appropriate predictors for clinical use [2]. Some recent studies have analyzed the possible diagnostic value of CT for discriminating between benign and malignant thyroid nodules [[4], [5], [6], [7]]. Although we have found a large amount of data used to analyze singular patterns of calcification as predictors of malignancy, even using radiological assessments, we have found no data that refer to a possibly higher malignancy risk related to the coexistence of multiple patterns of calcification [[8], [9], [10], [11]].

In cases such as ours that involve different patterns of calcification, which patterns should be regarded as more important for choosing the correct therapeutic procedure?

For nodules associated with different types of calcification, the pattern that is associated with a higher risk of a malignant prognosis, rather than simply the most represented pattern, should probably be regarded as the dominant type.

In our experience, although we encountered the extensive macrocalcification of a large nodule (6 cm) with a controversial pattern of calcifications, this nodule was eventually determined to be benign. We opted for the surgical removal of the thyroid gland merely due to its dimensions and the discomfort that the patient was suffering. However, no publications in the current literature provide a clear description of how a combined micro- and macrocalcification pattern is related to the risk of a malignant or benign lesion. Will it be possible in the future to better compare how radiological findings relate to nodule histology? Is an FNA biopsy indicated in cases involving combined calcification in the same nodule, even if a high risk of a false negative has been described? In a retrospective study, Zhou et al. [12] studied the quantitative contrast-enhanced US indicators for discriminating between benign and malignant thyroid nodules. The authors concluded that quantitative contrast-enhanced US may be useful and that the nodule to peri-nodule peak intensity ratio showed the best diagnostic efficacy; however, the situation remains unclear and controverted.

In summary, our case report highlighted some interesting points, which should be taken into consideration for further clinical study: 1. the impossibility of performing an FNA biopsy in some situations, as in our case, where the lobes were completely calcified not only inside but also externally; 2. the necessity of performing a thyroidectomy even without a biopsy, based on the symptoms of the patient and due to the low probability of obtaining a good quality sample, which is useful for the diagnosis; 3. despite the scientific literature regarding microcalcifications, which are mainly considered as a sign of papillary carcinoma, we showed a case of a completely calcified thyroid goiter (with micro- and macrocalcifications) with a benign diagnosis. This could suggest the necessity in the future to better define or reconsider the calcifications in a thyroid goiter, which of course is not possible to be determined by a single case, but in further multiple-case studies. We believe that attention should be focused on a better classification and definition of the calcifications, not only regarding the radiological findings, as recently described, but also in regard to discovering new approaches to analyzing their characteristics.

## Conflicts of interest

The Authors have no financial and personal relationships to disclose.

## Sources of funding

No funding.

## Ethical approval

For single case report NO ethical approval needs. Patient signed a consent for publishing the case report.

## Consent

Patient signed a consent for the publication of this case report.

## Author’s contribution

DB and BA wrote the case report. AS and UM revised the case report.

## Registration of research studies

Ethical Board approval is not required for case reports in our Center.

## Guarantor

Prof. Uliano Morandi is the Guarantor of this case report.

## Provenance and peer review

Not commissioned, externally peer-reviewed.

## References

[1] Kim Bu Kyung (2013). Relationship between patterns of calcification in thyroid nodules and histopathologic findings. Endocr. J..

[2] Na Dong Gyu (2016). Thyroid nodules with isolated macrocalcification: malignancy risk and diagnostic efficacy of fine-needle aspiration and core needle biopsy. Ultrasonography.

[3] Agha R.A., Borrelli M.R., Farwana R., Koshy K., Fowler A., Orgill D.P., For the SCARE Group (2018). The SCARE 2018 statement: updating consensus surgical CAse REport (SCARE) guidelines. Int. J. Surg..

[4] Yang T.-T., Huang Y., Jing X.-q. (2016). CT-detected solitary thyroid calcification: an important imaging feature for papillary carcinoma. OncoTargets Ther..

[5] Wu C.W., Dionigi G., Lee K.W. (2012). Calcifications in thyroid nodules identified on preoperative computed tomography: patterns and clinical significance. Surgery.

[6] Zhang L.-x., Xiang J.-j., Wei P.-y. (2018). Diagnostic value of computed tomography (CT) histogram analysis in thyroid benign solitary coarse calcification nodules. J. Zhejiang Univ. Sci. B.

[7] Gao S.Y., Zhang X.Y., Wei W. (2016). Identification of benign and malignant thyroid nodules by in vivo iodine concentration measurement using single-source dual energy CT: a retrospective diagnostic accuracy study. Medicine (Baltimore).

[8] Arpaci Dilek (2014). Evaluation of cytopathological findings in thyroid nodules with macrocalcification: macrocalcification is not innocent as it seems. Arq. Bras. Endocrinol. Metab..

[9] Park Yun Joo (2014). Thyroid nodules with macrocalcification: sonographic findings predictive of malignancy. Yonsei Med. J..

[10] Oh Eun Mee (2014). The pattern and significance of the calcifications of papillary thyroid microcarcinoma presented in preoperative neck ultrasonography. Ann. Surg. Treat. Res..

[11] Hoang Jenny K. (2007). US features of thyroid malignancy: pearls and pitfalls. RadioGraphics.

[12] Zhou X., Zhou P., Hu Z. (2018). Diagnostic efficiency of quantitative contrast-enhanced ultrasound indicators for discriminating benign from malignant solid thyroid nodules. J. Ultrasound Med..

